# Impact of COVID-19 on Tuberculosis Indicators in Brazil: A Time Series and Spatial Analysis Study

**DOI:** 10.3390/tropicalmed7090247

**Published:** 2022-09-14

**Authors:** Thaís Zamboni Berra, Antônio Carlos Vieira Ramos, Yan Mathias Alves, Reginaldo Bazon Vaz Tavares, Ariela Fehr Tartaro, Murilo César do Nascimento, Heriederson Sávio Dias Moura, Felipe Mendes Delpino, Débora de Almeida Soares, Ruan Víctor dos Santos Silva, Dulce Gomes, Aline Aparecida Monroe, Ricardo Alexandre Arcêncio

**Affiliations:** 1 Department of Maternal-Infant and Public Health Nursing, Ribeirão Preto College of Nursing, University of São Paulo, Ribeirão Preto 14040-902, SP, Brazil; 2 Mathematics Department, University of Évora, 7000-671 Évora, Portugal

**Keywords:** COVID-19, tuberculosis, health impact assessment, time series studies, interrupted time series analysis, ecological studies

## Abstract

Background: We aimed to visualize and classify the time series of COVID-19, tuberculosis (TB) notification, and TB outcomes (cure, treatment abandonment, and death), verify the impact of the new coronavirus pandemic on these indices in Brazil, and verify the presence of spatial autocorrelation between COVID-19 and TB. Methods: This was an ecological time series study that considered TB and COVID-19 cases. Seasonal Trend Decomposition using Loess (STL) was used to trace the temporal trend, Prais–Winsten was used to classify the temporal trend, Interrupted Time Series (ITS) was used to verify the impact of COVID-19 on TB rates, and the Bivariate Moran Index (Global and Local) was used to verify the spatial autocorrelation of events. Results: Brazil and its macro-regions showed an increasing temporal trend for the notification of TB in the pre-pandemic period. Only the Northeast Region showed a decreasing temporal trend for cured cases. For treatment abandonment, all regions except for the Northeast showed an increasing temporal trend, and regarding death, Brazil and the Northeast Region showed an increasing temporal trend. With the ITS, COVID-19 caused a decline in TB notification rates and TB outcome rates. With the global spatial analysis, it was possible to identify the existence of spatial autocorrelation between the notification rate of COVID-19 and the TB notification rate and deaths. With the local analysis, it was possible to map the Brazilian municipalities and classify them according to the relationship between the rates of both diseases and space. Conclusions: COVID-19 influenced the follow-up of and adherence to TB treatment and intensified social vulnerability and, consequently, affected the notification of TB since the relationship between the disease and social determinants of health is already known. The restoration and strengthening of essential services for the prevention and detection of cases and treatment of TB in endemic environments such as Brazil have been oriented as a priority in the global health agenda.

## 1. Background

The impacts of the new coronavirus are profound and have affected all spheres of global society, which can be seen in the consequences that afflict both the physical and psychosocial integrity of the population (including the death of close people), even in the socioeconomic sphere.

According to a report released by the World Health Organization (WHO), at the end of December 2019, the first case of severe pneumonia (caused by coronavirus 2 or SARS-CoV-2) was recorded in the city of Wuhan, Hubei Province, China, but it quickly spread all over the world [1]. On 20 March 2020, the transmission of COVID-19, the name given to the disease caused by the new coronavirus, was already happening throughout the Brazilian national territory, with the first case recorded in Brazil on 26 February 2020, which was two months after the first reported case in Wuhan, China [2].

With the COVID-19 pandemic declared, calamitous scenarios were intensified in several countries, especially issues related to public health in developing countries, further accentuating existing social inequalities and aggravating the situation of other diseases, such as tuberculosis (TB), which, in Brazil, became even more challenging in terms of its control and eradication in the midst of this scenario [3].

It is noteworthy that TB became the leading cause of death among infectious diseases in the world in 2015 when it overtook Human Immunodeficiency Virus (HIV) infection [4]. However, on 1 April 2020, COVID-19 overtook TB in death numbers per day, making it currently the deadliest infectious disease [5,6]. Data from the latest WHO global TB report showed an 18% reduction in the number of reported cases in 2020 compared to 2019 [7].

In 2020, according to the WHO, Brazil recorded almost 67,000 new cases of TB, with an incidence rate of 31.6 cases per 100,000 inhabitants, and in 2019, around 4500 deaths from the disease were recorded, with a mortality rate of 2.2 deaths per 100,000 inhabitants [8,9]. According to the Ministry of Health, from 26 February 2020 to 15 August 2022, 34,178,240 cases and 681,557 deaths from COVID-19 were confirmed in Brazil, with an incidence rate of 16,264 cases per 100,000 inhabitants and a mortality rate of 3243 deaths per 100,000 inhabitants [10].

Since a pandemic was declared for COVID-19, cases of TB and COVID-19 have been reported concomitantly, which is an association with great potential for morbidity and mortality [11,12]. It is noteworthy that high clinical suspicion is required for a diagnosis of TB in the era of COVID-19 since both diseases have similar symptoms, including fever and respiratory symptoms, especially persistent cough and shortness of breath [11,13].

As TB is a critical public health problem, the most evident consequence of the COVID-19 pandemic was the decrease in new diagnoses and notifications. This issue may be related to several factors, such as difficulty in accessing the health system, social isolation, and similarity between the symptoms of the two diseases, among others [14].

We performed a literature search on the subject and noted that there are still few studies on the impact of the COVID-19 pandemic on the notification and outcomes of TB cases, especially in Brazil. A recent study [15] used data from 33 centers in 16 countries on 5 continents and showed that care in TB referral units was lower during the first 4 months of the pandemic in 2020 than in the same period in 2019 and clearly indicated that the rates of active and latent TB diagnoses declined during the COVID-19 pandemic in many countries. Another study [16], this one carried out in Brazil, found that the number of new TB cases in the state of Bahia was 26.4% lower in the period from January to July 2020 than in the same period in 2019.

Thus, the present study aimed to visualize and classify the time series of COVID-19, TB, and TB outcomes (cure, treatment abandonment, and death), verify the impact of the new coronavirus pandemic on these indices in Brazil, and verify the presence of spatial autocorrelation between COVID-19 and TB.

## 2. Methods

### 2.1. Study Design and Scenario

We performed an ecological [17] time series study in Brazil and its macro-regions. Brazil is in South America and has a territorial extension of 855,767 km^2^ and an estimated population of 208.4 million, distributed in five macro-regions (North, Northeast, South, Southeast and Midwest) [18].

### 2.2. Population

All reported TB cases from January 2010 to December 2021 (1,054,793 cases) and all reported cases of COVID-19 from February 2020 to December 2021 (22,287,521 cases) were included in this study.

Information regarding TB cases was obtained through the Department of Informatics of the Unified Health System (DATASUS), information regarding COVID-19 cases was obtained from the Coronavirus Panel, and population data were extracted from the Brazilian Institute of Geography and Statistics (IBGE).

### 2.3. Analysis Plan

Initially, the monthly TB notification rates were calculated for the period from January 2010 to December 2021, and those for COVID-19 were determined for February 2020 (when the first notification of the disease occurred in the national territory) to December 2021; the numerator was the number of cases, and the denominator was the population and multiplication factor per 100,000 inhabitants.

TB cases were grouped according to the outcomes into cured, treatment abandonment, or death, and other outcomes were not considered in the study. As defined by the Ministry of Health, the cured outcome occurs when the person with TB performs the complete treatment regimen and presents two negative smears, one in the follow-up phase and the other at the end of treatment. Treatment abandonment occurs when the person with TB fails to attend the health unit that is carrying out their follow-up for more than 30 consecutive days after the scheduled date for their return (in cases of supervised treatment, the period of 30 days starts from the date of the last medication intake since one of the objectives of the home visit carried out by the health team is to prevent the patient from abandoning treatment). Finally, death is when TB is the declared underlying cause, regardless of whether or not the person was being treated for TB [19].

To verify the behavior of the time series of these outcomes, their monthly rates were also calculated for the period from January 2010 to December 2021, with the number of cases with a given outcome as the numerator and the population and multiplication factor per 100,000 inhabitants as the denominator.

### 2.4. Time Series Analysis

It is noteworthy that time series are characterized as a set of observations obtained sequentially over time [20]. Thus, monthly time series of previously calculated rates of COVID-19, TB, and TB outcomes were constructed. To verify the behavior of the time series over the period under study and to verify its temporal trends, we used the decomposition method called Seasonal Trend Decomposition using Loess (STL), which is based on locally weighted regression [21].

### 2.5. Classification of Time Trend

To classify and verify the temporal trends of TB and its outcomes in Brazil and its macro-regions in the pre-COVID-19 pandemic period, we considered the period from 2010 to 2019, and the previously calculated rates were logarithmized (log10) to reduce and stabilize the variance effect of the data [20].

The Prais–Winsten autoregression method was performed to classify the temporal trend of events as increasing, decreasing, or stationary in the study period.

When the temporal trend was classified as increasing or decreasing, the percentages of monthly variation (MPC—monthly percent change) and their respective 95% confidence intervals (95%CI) were calculated [20].

### 2.6. Impact of COVID-19

To verify whether the COVID-19 pandemic had any impact on the temporal trend of TB rates and its outcomes, the technique called Interrupted Time Series (ITS) was used, which is described as the most effective technique to assess the impact of an intervention, with two parameters defining each segment of the series: level and trend [22].

In the present study, the COVID-19 pandemic is considered an intervention; the level is considered the initial value of the series in each segment and indicates whether the intervention caused a break in the time series, that is, an abrupt change in the behavior of the time series, either increasing or decreasing its indices, which can then be considered. A gradual change in the values of the time series after the beginning of the intervention is considered a trend [22].

In summary, the objective of this technique is to assess whether there is an immediate impact (change in level) and/or progressive impact (change in trend) on the values of the series when an intervention occurs, for which we call the level “intervention” and the progressive impact caused by COVID-19 on TB rates “post-intervention”.

The first case of COVID-19 registered in Brazil occurred on 27 February 2020 in the state of São Paulo in the Southeast Region of the country, and therefore, the month of February 2020 was the cut-off point in the study to determine whether, after the arrival of the new coronavirus in Brazil and the beginning of the COVID-19 pandemic, there was a change in the notification rates of TB and its outcomes in Brazil and its macro-regions.

### 2.7. Spatial Analysis

For this stage of the study, we decided to use all 5568 Brazilian municipalities [18] as the unit of analysis, for which the notification rates of COVID-19, TB, and their outcomes were calculated between the years 2020 and 2021, with the number of cases in the numerator and the population and the multiplication factor per 1000 inhabitants in the denominator.

The Global and Local Bivariate Moran Indexes [23] were used to verify the spatial autocorrelation of COVID-19 notification rates with the TB indices analyzed in the present study (notification rates, cure, treatment abandonment, and death).

The Global Bivariate Moran Index verifies whether a variable observed in a region has spatial dependence (positive or negative) with another variable of interest [23].

On the other hand, the Local Bivariate Moran Index indicates the degree of association (positive or negative) between the value of the variable of interest in a given region and another variable in the same region, and in this way, it is possible to map the statistically significant values, generating a choropleth map according to its classification, always relating the values of the variable of interest in a given region to the values of the other variable in the same region: High–High (high values of the first variable with high values of the second variable), Low–Low (low values of the first variable with low values of the second variable), Low–High (low values of the first variable with high values of the second variable), and High–Low (high values of the first variable with low values of the second variable). It is noteworthy that the high and low values are classified according to the average values of the variables in the neighboring regions [23].

### 2.8. Statistical Analysis

We calculated the TB and COVID notification rates and TB outcomes as well as their logarithmization (log10) using the Microsoft Office Professional Plus 2016 software (Microsoft Corporation, Redmond, Washington, DC, USA), specifically Excel spreadsheets. STL was performed using the forecast package [24] in Rstudio software version 4.0.3 (RStudio Team, 250 Northern Ave, Boston, MA, USA). Finally, the Prais–Winsten autoregression method and the STI method were performed using the STATA version 14 software (StataCorp LLC, College Station, TX, USA).

In the spatial analysis stage, the Global and Local Bivariate Moran Indexes were analyzed using the free software GeoDa. Shapefiles with the results of the analysis were exported, and the final map was prepared using ArcGis software version 10.5 (Esri, Redlands, CA, USA).

## 3. Results

Between 2020 and 2021, Brazil recorded 22,287,521 cases of COVID-19: 8.7% in the North Region, 22.2% in the Northeast Region, 19.5% in the South Region, 38.9% in the Southeast Region, and 10.7% in the Midwest Region of the country.

**Figure 1:**
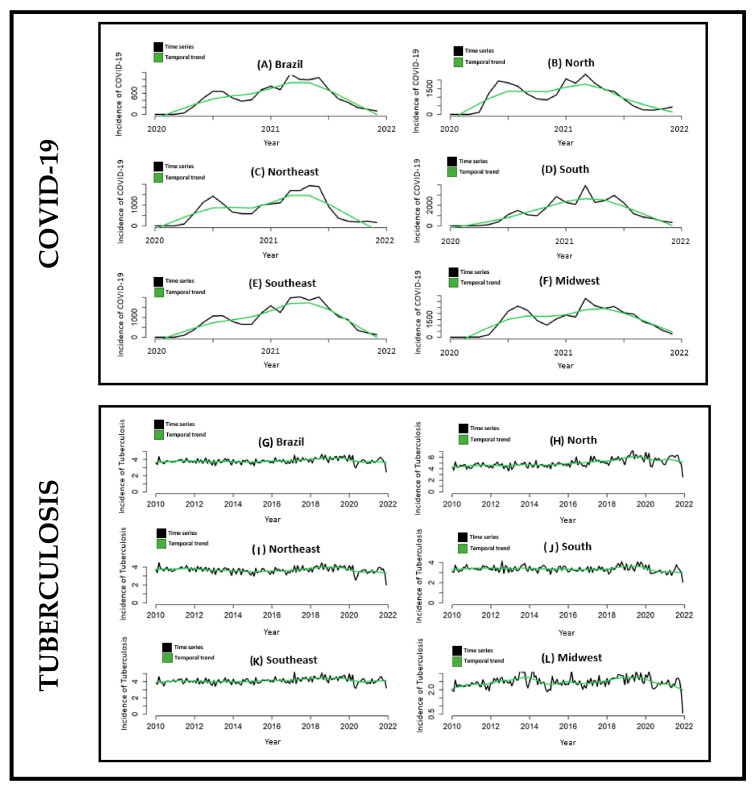
Monthly time series and temporal trend of the notification of COVID-19 and tuberculosis in Brazil and its macro-regions (2020–2021). Legend: (**A**)-Notification rate (black line) and temporal trend (green line) of COVID-19 in Brazil. (**B**)-Notification rate (black line) and temporal trend (green line) of COVID-19 in the North Region of Brazil. (**C**)-Notification rate (black line) and temporal trend (green line) of COVID-19 in the Northeast Region of Brazil. (**D**)-Notification rate (black line) and temporal trend (green line) of COVID-19 in the South Region of Brazil. (**E**)-Notification rate (black line) and temporal trend (green line) of COVID-19 in the Southeast Region of Brazil. (**F**)-Notification rate (black line) and temporal trend (green line) of COVID-19 in the Midwest Region of Brazil. (**G**)-Notification rate (black line) and time trend (green line) of Tuberculosis in Brazil. (**H**)-Notification rate (black line) and time trend (green line) of Tuberculosis in North Region of Brazil. (**I**)-Notification rate (black line) and temporal trend (green line) of Tuberculosis in the Northeast Region of Brazil. (**J**)-Notification rate (black line) and time trend (green line) of Tuberculosis in South Region of Brazil. (**K**)-Notification rate (black line) and temporal trend (green line) of Tuberculosis in the Southeast Region of Brazil. (**L**)-Notification rate (black line) and time trend (green line) of Tuberculosis in the Midwest Region of Brazil.

The time series of notification rates and the time trend of COVID-19 in Brazil and its macro-regions can be seen in Figure 1A–F, which clearly visualizes the two main waves of the disease recorded in the country, the first being in mid-2020 and the second in mid-2021. It is noteworthy that all regions of the country behaved similarly, with only the South Region registering a different behavior from the others.

Regarding TB, between 2010 and 2021, Brazil recorded 1,054,793 cases of the disease: 10.8% in the North Region, 26.5% in the Northeast Region, 12.5% in the South region, 45.2% in the Southeast Region and 4.7% in the Midwest Region of the country.

The time series of TB incidence rates in Brazil and its macro-regions can be seen in Figure 1G–L, which shows that the highest rates of the disease were recorded in the North Region, and the lowest rates were recorded in the Midwest Region of the country, where the registered indices were the only ones below the national indices.

Between 2010 and 2021, Brazil recorded 674,978 cured TB cases (2.1% North, 28.3% Northeast, 13.8% South, 48.9% Southeast, and 6.5% Midwest), 127,932 cases of treatment abandonment (10.4% North, 24.3% Northeast, 13.3% South, 47.4% Southeast, and 4.4% Midwest), and 36,997 cases of death from the disease (7.9% North, 28.9% Northeast, 12.6% South, 46.1% Southeast, and 4.3% Midwest).

**Figure 2:**
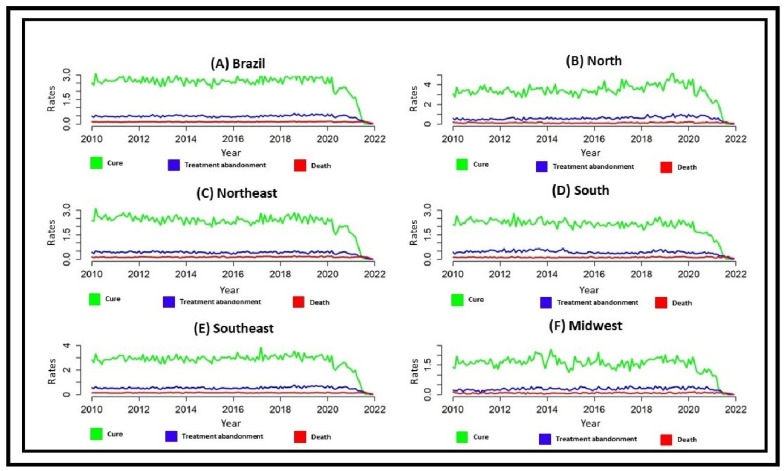
Monthly time series of tuberculosis outcomes in Brazil and its macro-regions (2010–2021). Legend: Cure—when a person with TB undergoes the complete treatment regimen and presents two negative smears, one in the follow-up phase and the other at the end of treatment. Treatment abandonment—when a person with TB fails to attend the health unit or fails to take the medication for more than 30 consecutive days. Death—when TB is the declared underlying cause, regardless of whether or not the person was being treated for TB. (**A**)-Outcome rate of tuberculosis cases reported in Brazil (green line = cure, blue line = treatment abandonment, red line = death). (**B**)-Outcome rate of tuberculosis cases reported in North Region of Brazil (green line = cure, blue line = treatment abandonment, red line = death). (**C**)-Outcome rate of tuberculosis cases reported in the Northeast Region of Brazil (green line = cure, blue line = treatment abandonment, red line = death). (**D**)-Outcome rate of tuberculosis cases reported in South Region of Brazil (green line = cure, blue line = treatment abandonment, red line = death). (**E**)-Outcome rate of tuberculosis cases reported in the Southeast Region of Brazil (green line = cure, blue line = treatment abandonment, red line = death). (**F**)-Outcome rate of tuberculosis cases reported in the Midwest Region of Brazil (green line = cure, blue line = treatment dropout, red line = death).

Figure 2 presents the time series of the mentioned outcomes. Regarding cure and treatment abandonment, the highest and lowest rates were recorded, respectively, in the North and Midwest Regions of the country, while for deaths, all regions showed similar behavior to the national rates, except the Midwest Region, which maintained the lowest rates throughout the study period.

With the Prais–Winsten auto-regression technique, it was possible to classify the temporal trend of TB notification and its outcomes in Brazil and its macro-regions in the pre-COVID-19 pandemic period.

Brazil and all of its macro-regions showed an increasing temporal trend for the notification of TB in the period between 2010 and 2019. Regarding the outcomes, only the Northeast Region of Brazil showed a decreasing temporal trend (−0.11%/month; 95%CI: −0.18–−0.05) for the cured group, while the other regions showed a stationary trend. For the outcome of treatment abandonment, with the exception of the Northeast Region, all of the others showed an increasing temporal trend. Finally, regarding death, Brazil and the Northeast Region showed increasing temporal trend of +0.02%/month (95%CI: 0.01–0.04) and +0.18%/month (95%CI: 0.09–0.28), respectively, while the other regions showed a stationary trend.

Table 1 presents the classifications of temporal trends in Brazil and its macro-regions and the percentage of monthly variation in TB notification and outcomes.

With the application of ITS, as shown in Table 2, it was possible to verify that, in Brazil, COVID-19 caused a decline in the TB incidence rate and also in the cure rate. After the arrival of COVID-19 in the country, the cure category had a decreasing temporal trend of −15.02%/month (95%CI: −25.50–−46.21), treatment abandonment decreased at −1.98%/month (95%CI: −31.98–−34.65), and death decreased at −15.35%/month (95%CI: −3.17–−0.64).

In the North Region, there was also a break in the series, with a decline in notification rates, a decrease in cured cases of −18.78%/month (95%CI: −21.85–−15.59), and a decrease in treatment abandonment of −10.97%/month (95%CI: −12.76–−9.19). The Northeast Region also showed a break in the time series, with a decline in notification and cure rates, and in the post-intervention period, there was a decrease of −22.07%/month (95%CI: −26.36–−17.52) for cured cases, −3.15%/month (95%CI: −5.74–−0.49) for treatment abandonment, and −5.62%/month (95%CI: −8.27–−2.89) for death.

The South, Southeast, and Midwest Regions of Brazil also showed breaks, with declines in their time series of TB notification and cured cases. In the post-intervention period, that is, after the arrival of the new coronavirus and the beginning of the pandemic in the country, the South Region showed a decreasing trend for cured cases (−19.52%/month; 95%CI: −15.15–−23.67) and treatment abandonment rates (−14.03%/month; 95%CI: −9.75–−18.09).

In the same period, the Southeast Region showed a decrease of −21.62%/month (95%CI: −16.36–−26.54) for cured cases, −16.11%/month (95%CI: −11.04–−20.89) for treatment abandonment, and −2.63%/month (95%CI: −1.64–−3.60) for death. Finally, also in the post-intervention period, the Midwest Region showed a decrease of −2.73 (95%CI: −1.19–−4.25) for the notification rates and a decrease of −18.91%/month (95%CI: −03.22–−15.66) for cured cases of TB.

With the application of the Global Bivariate Moran Index, it was possible to identify spatial autocorrelations between the notification rate of COVID-19 and the notification rate of TB (−0.004; *p*: 0.01) and death from TB (−0.004; *p*: 0.01), which both presented a negative correlation index, that is, high rates of notification of COVID-19 were spatially autocorrelated with low rates of notification and death from TB.

**Figure 3:**
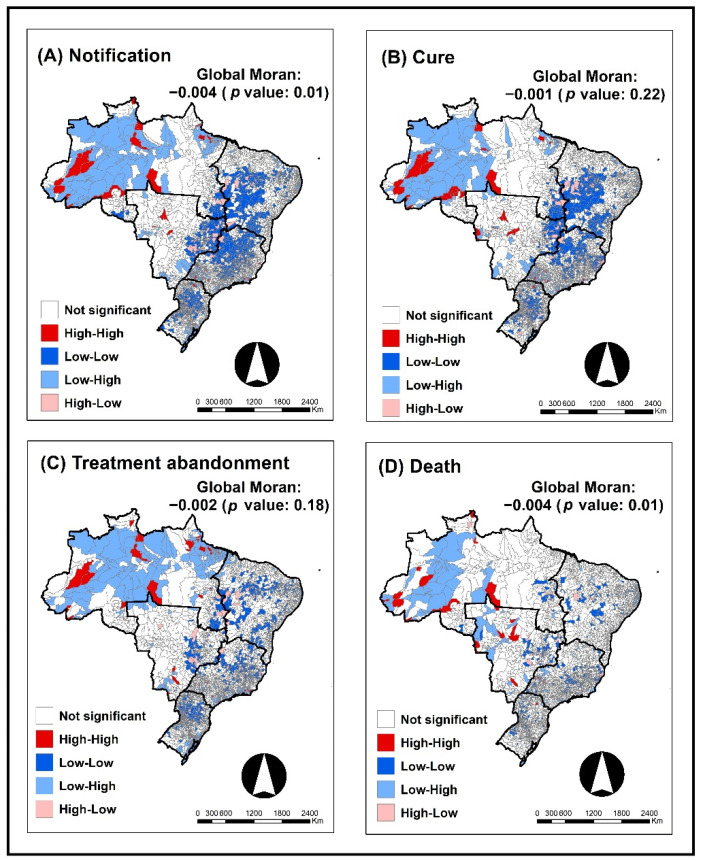
Analysis of bivariate spatial autocorrelation between the notification rate of COVID-19 and notification of TB and its outcome (cure, treatment abandonment, and death) according to Brazilian municipalities (2020–2021). (**A**)-Application of the Global and Local Bivariate Moran technique between reported cases of COVID-19 and reported cases of tuberculosis. (**B**)-Application of the Global and Local Bivariate Moran technique between reported cases of COVID-19 and cured tuberculosis cases. (**C**)-Application of the Global and Local Bivariate Moran technique between reported cases of COVID-19 and tuberculosis cases that abandoned treatment. (**D**)-Application of the Global and Local Bivariate Moran technique between reported cases of COVID-19 and tuberculosis cases that had death as an outcome.

Figure 3 shows that with the application of the Local Bivariate Moran Index, in which a relationship with TB notification is noted (Figure 3A), in general, most municipalities in the Northern Region of the country were classified as Low–High, that is, the presence of low rates of COVID-19 and high rates of TB, but there were also municipalities classified as High–High (high rates of COVID with high rates of TB). In the other regions of the country, the predominant category for TB notification was Low–Low, with some municipalities classified as Low–High or High–Low, a pattern similar to that observed for the relationship between the notification rate of COVID-19 and the TB cure rate (Figure 3B).

Regarding treatment abandonment (Figure 3C), the region of Brazil that stands out the most is the North Region, with most municipalities classified as Low–High and some classified as High–High. The rest of the country has, for the most part, municipalities classified as Low–Low and a few classified as High–High or High–Low.

Finally, regarding death (Figure 3D), most of the statistically significant municipalities belong to the North Region of the country, with a predominance of the Low–High classification and the presence of High–High and Low–Low municipalities. In the Midwest Region of Brazil, the presence of High–High municipalities stands out, and in the Northeast and Southeast Regions, there is a predominance of Low–Low municipalities, but some municipalities are classified as High–Low.

## 4. Discussion

This study aimed to visualize and classify the time series of COVID-19, TB, and TB outcomes (cure, treatment abandonment, and death), verify the impact of the new coronavirus pandemic on these indices in Brazil, and verify the presence of spatial autocorrelation between COVID-19 and TB.

In the period from 2020 to 2021, Brazil recorded 22,287,521 cases of COVID-19, ranking third among nations that recorded the disease throughout the world [1]. In Brazil, the measures adopted since 2020 by federal, state, and municipal executives, such as physical distancing, expansion of hospital beds, and implementation of surveillance systems, did not prevent the uncontrolled spread of the pandemic or the appearance of two large waves of transmission between 2020 and 2021 at breakneck speed [25].

From the graphs presented in Figure 1 referring to the time series of the notification rates and the temporal trend of COVID-19 in Brazil and its macro-regions, it is possible to clearly visualize these two waves of the disease that the country recorded, the first being in mid-2020 and the second in mid-2021.

Of the complications related to the new coronavirus, coinfections stand out, which have been a reality for people affected by COVID-19, especially for those with severe involvement, including fungal, viral, and bacterial infections, but especially TB. It is estimated that a quarter of the world population (approximately 2 billion people) is infected with TB (latent TB), and to date (July 2022), there are about 554 million confirmed cases of TB–COVID-19 coinfection [1,26].

According to the WHO, there were about 1.4 million fewer cases of undetected and untreated TB in 2020, a 21% decrease compared to 2019 [27]. In Brazil, in 2020, the average number of reported TB cases decreased by about 6.5 cases compared to the period from 2017 to 2019, with the exception of the Northern Region of the country [28]. Currently, the COVID-19 pandemic is identified as one of the main obstacles to the effectiveness of TB control measures in the world in the last two years [28,29].

Previous studies have shown that the immune status that enhances vulnerability to TB can also contribute to susceptibility to coronavirus infection [30,31]. TB and COVID-19, two diseases with different historical contexts, have great similarities; for example, the transmission mechanism of both is airborne, the main symptoms include cough and fever, they have great potential for pulmonary structural sequelae, and they are permeated by social stigma [32].

In a case study published by Italian authors [33], a patient with in vitro immune cell anergy affected by bilateral cavitary pulmonary TB and COVID-19-associated pneumonia was described. The patient died 13 days after the diagnosis of TB, and it is hypothesized that the combined effect of TB and COVID-19 infections probably caused pronounced lymphocytopenia [33,34], which is a reliable indicator of the severity and hospitalization of COVID-19 patients [34], and, consequently, caused a decrease in CD4 + cells, as described in coinfections with COVID-19 [35] and TB and SARS [36]. Furthermore, COVID-19 was the precipitating factor of TB respiratory failure, where a possible cause for this deterioration is the immunosuppression of the patient with advanced TB, together with the possible compromise of the immune system due to SARS-CoV-2 [33].

The pathogenicity of COVID-19 is still unknown, and reports of concomitant TB are quite limited. However, the literature shows that certain viral infections, such as measles, can exacerbate pulmonary TB, probably because of depressed cellular immunity [33,37] since TB normally requires cellular immunity, particularly CD4-mediated immunity [38,39], in addition to the patient being transiently immunosuppressive.

Another study [40] found that advanced age, comorbidities such as hypertension, hepatitis, and cancer, symptoms of dyspnea on admission, CT imaging characteristics of bilateral lung lesions, infiltrates, and tree-in-bud, and higher leukocyte count can be predictors of poor prognosis in patients with COVID-19–TB. In this way, it emphasizes the importance of performing or repeating tests for COVID-19 to obtain a reliable and early result based on a clinical suspicion of COVID-19 in a patient with pulmonary TB so that immediate isolation precautions and specific treatment are carried out, especially in groups considered at risk.

Thus, it is also suggested that professionals be aware of the possibility of pneumonia associated with COVID-19 in the differential diagnosis of patients with active TB. This can be challenging since, as mentioned earlier, both diseases share many similarities both in symptoms and in their radiological aspects, which can be confused with one another, so COVID-19 can be a risk factor for death, especially in a fragile population such as TB patients [33].

In Brazil, in the period from 2010 to 2019 (prior to the COVID-19 pandemic), the country and all of its macro-regions showed an increasing temporal trend of the TB notification rate, with an emphasis on the Northern Region of Brazil, which historically has the highest TB burden in the country, and conversely, the South region, which has the lowest indicators of the disease (Figure 2).

According to data released by the WHO, among the 30 countries where 84% of TB cases are concentrated, there was a 21% drop in TB notifications when comparing data from 2019 and 2020, and it is estimated that this drop could generate half a million additional TB deaths. In Brazil, when comparing data from 2019 with 2020, there was a 10.9% drop in TB notifications, with a drop after April and the greatest effect observed in May with 31.9% of cases, which was a reduction when compared to the same period in 2019. Among the regions, the smallest drop in the evaluated period was observed in the Southeast Region (−9.4%), and the largest occurred in the South Region. The drop in the notification rate and the consequent search for new TB cases raises an alert for the need to evaluate and adapt TB control actions in the country, aiming at timely decision making by managers and health professionals involved in the control of the disease [26,41].

With the application of the Local Bivariate Moran Index, it was possible to identify municipalities that deserve attention from municipal, state, and federal managers, since studies suggest that TB and COVID-19 are considered a “cursed duet” with a poor prognosis, and thus, these people need immediate attention. TB should be considered a risk factor for severe COVID-19 disease, and TB patients should be prioritized for COVID-19 prevention efforts, including vaccination [13,40,42].

A meta-analysis study [40] indicated that people with COVID-19–TB coinfection are more than twice as likely to die or develop serious complications than patients with COVID-19 or TB alone, respectively. Thus, it is important to perform routine screening for the detection of *Mycobacterium tuberculosis* among suspected or confirmed cases of COVID-19 in places already known to have a high TB burden due to the worse prognosis of this coinfection and the very similar clinical symptoms between the diseases.

Brazil is a country of continental dimensions, which presents a wide climatic and especially socioeconomic diversity, factors that affect the transmissibility and magnitude (impact) of TB. In 2015, Brazil was classified as a country with a high level of general human development, occupying the 79th position in the world rankings of countries by the Human Development Index (HDI) [43], although this level was classified as low in the North and Northeast Regions (highly endemic regions for TB) and higher in the South and Midwest Regions (where the notification rate was lower).

In an investigation carried out in Brazil that analyzed the temporal trend of TB in the period from 2006 to 2015, it was identified that the highest notification rates of the disease were recorded in the North Region of the country, while the lowest rates were identified in the South and Midwest Regions of Brazil [44]. Demographic heterogeneity is a factor that influences the incidence and mortality associated with TB in Brazil, and the transmission of the disease is closely associated with agglomeration, as observed in the metropolitan areas of Brazil [44,45], with the regions with the highest numbers of reported cases in the Southeast and Northeast macro-regions, respectively, which are the most populous in the country [44].

A previous study [28] that analyzed the effect of the COVID-19 pandemic on the burden of TB incidence across regions of Brazil showed the lowest incidence rates of the disease in the South and Midwest Regions of Brazil. Although the South and Midwest Regions have high human development indices (HDIs), they have very different characteristics when the social and health contexts are analyzed.

Regarding cured cases and treatment abandonment, it is noted that the highest and lowest rates were recorded in the North and Midwest Regions of the country, respectively, while for death, all regions showed similar behavior to the national rates except the Midwest Region, which maintained the lowest rates throughout the study period.

Regarding treatment abandonment, a literature review [46] showed that the main risk factors for not completing treatment for TB are being male, being aged between 30 and 39 years, being illiterate or having low education, using drugs, having undergone previous treatment for TB, living far from health services, and having chronic diseases such as diabetes and HIV.

In this way, health professionals should seek to know and understand the perspective of the person with TB on adherence and their behaviors, since the interaction between the patient and professional is an essential part of care. The person with TB should be guided regarding their diagnosis, the method used to arrive at it, reasons for carrying out the treatment, potential adverse reactions, and consequences of treatment irregularity, thus becoming the protagonist of their care.

The Southern Region of Brazil has one of the highest percentages of primary care coverage in Brazil, which may be directly related to the effectiveness of TB prevention and control actions carried out in this region [47]. However, previous studies have shown that the Midwest Region has a percentage of primary care coverage below the standards recommended by the Ministry of Health, in addition to being a region characterized by having a high rural and indigenous population density [47,48].

In view of this, it can be inferred that the low rates of disease records in the Midwest Region may be related to the underreporting of new TB cases and segments performed and to the coverage of primary care services in that region, contrary to what happens in the South Region of Brazil.

The Northern Region of Brazil is characterized by low coverage of primary care in the Amazon territory, which is directly reflected in health indicators in this region—considered the worst indicators in Brazil [49]. In the case of TB, structural deficiencies in the provision of primary care services in the Amazon, combined with territorial distances and populations with greater social vulnerability and risk that inhabit that territory, can contribute to unfavorable outcomes of the disease since the implementation of strategies of diagnosis and clinical follow-up of TB is considered a constant challenge in that region [50].

Mortality from TB can be influenced by the relationship between the epidemiological surveillance of the disease and the quality of health actions offered, especially in primary care services, to a given population [51,52]. A previous study [52] showed that from 1990 to 2015, there was a significant decrease in TB mortality rates in Brazil. When analyzing the macro-regions, the Southeast presented the most significant percentage decrease in TB mortality rates, and the Northeast presented the lowest percentage of decrease.

Given this national panorama, it is inferred that, despite the advances observed in TB control in recent years, the temporal behavior of TB mortality rates observed during the study period confirms that TB is perpetuated as a serious public health problem in all regions of Brazil.

From this perspective, the immediate consequence of the COVID-19 pandemic was the reduction in new diagnoses/notifications, as observed in the present study using the ITS technique. The previously growing number of TB notifications in the world between 2017 and 2019 was followed by an 18% reduction in the interval between 2019 and 2020 (from 7.1 million to 5.8 million), with Brazil among the countries that contributed the most to this reduction. Concurrent with this scenario, there was an increase in TB deaths on global, regional, and national bases, reversing years of progress in reducing the number of deaths from the disease [26].

Even with the ITS technique, the cured cases of TB showed a decreasing temporal trend in all macro-regions of Brazil, while for treatment abandonment, only the Midwest Region showed a stationary trend, and the others also showed a decreasing temporal trend. Finally, for the outcome of death, the North, Northeast, and Southeast Regions showed a decreasing trend.

This decreasing trend deserves to be highlighted because, rather than indicating the success of policies and strategies to fight TB, it can be a contrary indicator and indicate that new cases are not being diagnosed and/or reported [53], as advised by the WHO in the TB 2020 report [26]. Therefore, the question arises of whether this decreasing trend is real or a reflection of underdetection and/or underreporting of cases, so local epidemiological surveillance teams must pay attention and seek to identify situations in which the reported data may differ from the true behavior of the disease [53].

An example of the importance of the health system’s responsiveness to TB and COVID-19 can be seen in a time series study [54] of TB diagnostic services in Mozambique. In that nation, the most significant impact occurred in the first quarter after the onset of COVID-19, with an abrupt 15% drop in the incidence of pulmonary TB, and, despite the initial impact, by the end of 2020, clear signs of recovery were already visible.

The COVID-19 pandemic has brought challenges to several sectors, including epidemiological surveillance, where improvements are needed in public policies aimed at reducing inequalities in access to health systems and strategies for a safe recovery, with the maintenance of actions and priority services, especially essential health programs such as the TB Control Program, in addition to the necessary reorganization of services previously intended for TB for the care of COVID-19 cases and the lack of personal protective equipment (PPE) and training for health professionals in relation to the differential diagnosis of TB and COVID-19 [3,55,56].

Under pressure from the growing number of COVID-19 cases, hospitals and health services were reorganized, resulting in the conversion of tuberculosis wards into COVID-19 units in many cases, which had a major impact on the care of TB patients and may be one of the explanatory factors for the reduction in TB rates. The impact of the COVID-19 pandemic on TB services has been estimated to be catastrophic, especially in regions where healthcare professionals involved in TB treatment have been transferred to manage the COVID-19 emergency [57,58,59], and a delay in diagnosis, more severe clinical presentations, worse outcomes, and loss of follow-up are just some of the indirect effects of the interruption of health services expected, mainly during the acute phases of the pandemic [15,60].

In addition, it is noteworthy that, in most regions, states, and municipalities, in addition to the paralysis of TB services, there was a need to redirect human resources to the front line against COVID-19, which directly affected the operationalization of DOT and, consequently, the active search for respiratory symptoms, which is also compromised by social distancing.

According to a recently published scoping review [3], all of these issues end up having a negative impact on the early diagnosis of TB cases, disease indicators, the increase in drug-resistant strains, treatment adherence, and the monitoring of cases and their communicators.

A study demonstrated a reduction in hospitalizations for TB, with a significant increase in the time of diagnosis during the period of 2020 when compared to 2019, in addition to the greater severity of clinical presentations that was observed in 2020 [57]. Other studies also showed an increase in the time of diagnosis even for non-communicable diseases [61,62,63], as people with acute and chronic conditions or those who had mild symptoms may have been discouraged from seeking medical care to reduce crowding in health facilities or due to the fear of becoming infected by COVID-19.

Early identification followed by the correct and complete treatment of people with TB are the main pillars for the prevention and control of the disease and should not stop as a result of the COVID-19 pandemic since all achievements and improvements in TB rates can go backwards; in addition, the relationship between TB and social vulnerability has been aggravated worldwide in the face of the new coronavirus, which has also had a great impact on the economic sphere.

## 5. Conclusions

The main limitation of this study is in its ecological design, which does not allow the generalization of its results to the individual level [17]. Another limitation of the work is the use of secondary data, which were sometimes unavailable or incomplete. Finally, the time series rates were calculated from the last Demographic Census, carried out in 2010, which, due to its lag, may not fully reflect the size of the population in the investigation period.

From the results presented, in the analyzed period, it was possible to verify the impact caused by the COVID-19 pandemic on TB rates and its outcomes in Brazil and its macro-regions. Given the protective measures adopted, COVID-19 influenced the follow-up of and adherence to TB treatment in addition to intensified social vulnerability and, consequently, affected the notification of TB since the relationship between the diseases and the social determinants of health is already known.

Thus, it is necessary that health services be reorganized so that they can meet the demands related to COVID-19 but also test suspected cases for TB and train health professionals so that they critically look at the suspicion of both diseases and remember that coinfection is possible. Thus, with the results of the present study, an even greater concern arises regarding the impact of the COVID-19 pandemic not only on TB rates but also on infectious diseases in general, for which improvement and elaboration of public policies are aimed at access to health services and monitoring throughout treatment.

It should be emphasized that delays in diagnosis can lead to increased transmission of *M. tuberculosis*. The largest effects of the COVID-19 pandemic are not yet all visible; therefore, rigorous monitoring involving all national TB centers must be carried out to quantify and prevent them. Faced with this worrying estimate, the restoration and strengthening of essential services for the prevention and detection of cases and treatment of TB in endemic environments, such as Brazil, have been oriented as a priority in the global health agenda.

## Figures and Tables

**Table 1:** Classification of the temporal trend of tuberculosis and its outcomes in Brazil and its macro-regions, according to the Prais-Winsten autoregression technique, in the pre-COVID-19 pandemic period (2010–2019).

Rate	Prais–Winsten (95%CI)	Trend	MPC (95%CI)
**Brazil**			
Notification	<0.01 (0.00–0.01)	Increasing	+0.04 (0.01–0.09)
Cure	<−0.01 (0.00–0.01)	Stationary	NA
Treatment abandonment	<0.01 (0.00–0.01)	Increasing	+0.06 (0.02–0.13)
Death	<0.01 (0.00–0.01)	Increasing	+0.02 (0.01–0.04)
**North**			
Notification	<0.01 (0.00–0.01)	Increasing	+0.23 (0.17–0.28)
Cure	<0.01 (0.00–0.01)	Stationary	NA
Treatment abandonment	<0.01 (0.00–0.01)	Increasing	+0.37 (0.37–0.52)
Death	<0.01 (0.00–0.01)	Stationary	NA
**Northeast**			
Notification	<−0.01 (0.00–0.01)	Stationary	NA
Cure	<−0.01 (0.00–−0.01)	Decreasing	−0.11 (−0.05–−0.18)
Treatment abandonment	<−0.01 (0.00–0.01)	Stationary	NA
Death	<0.01 (0.00–0.01)	Increasing	+0.18 (0.09–0.28)
**South**			
Notification	<−0.01 (0.00–0.01)	Stationary	NA
Cure	<−0.01 (0.00–−0.01)	Decreasing	−0.16 (−0.09–−0.22)
Treatment abandonment	<−0.01 (0.00–−0.01)	Decreasing	−0.16 (−0.03–−0.29)
Death	<−0.01 (0.00–0.01)	Stationary	NA
**Southeast**			
Notification	<0.01 (0.00–0.01)	Increasing	+0.06 (0.01–0.19)
Cure	<−0.01 (0.00–0.01)	Stationary	NA
Treatment abandonment	<0.01 (0.00–0.01)	Increasing	+0.13 (0.01–0.15)
Death	<−0.01 (0.00–0.01)	Stationary	NA
**Midwest**			
Notification	<0.01 (0.00–0.01)	Increasing	+0.07 (0.01–0.15)
Cure	<−0.01 (0.00–0.01)	Stationary	NA
Treatment abandonment	<0.01 (0.00–0.01)	Increasing	+0.32 (0.22–0.44)
Death	<0.01 (0.00–0.01)	Increasing	+0.25 (0.09–0.44)

Legend: 95%CI: 95% confidence interval; MPC: monthly percent change.

**Table 2:** Impact of COVID-19 on the temporal trend of tuberculosis and outcomes in Brazil and its macro-regions (2010–2021).

Rate	Intervention		Post-Intervention	
	Trend	MPC (95%CI)	Trend	MPC (95%CI)
**Brazil**				
Notification	Decreasing	−8.10 (−15.08–−0.54)	Stationary	NA
Cure	Decreasing	−1.98 (−0.54–5.96)	Decreasing	−15.02 (−25.50–−46.21)
Treatment abandonment	Stationary	NA	Decreasing	−1.98 (−31.98–−34.65)
Death	Stationary	NA	Decreasing	−15.35 (−3.17–−30.64)
**North**				
Notification	Decreasing	−8.47 (−6.21–−12.59)	Stationary	NA
Cure	Decreasing	−71.04 (−50.23–−83.15)	Decreasing	−18.78 (−15.59–−21.85)
Treatment abandonment	Decreasing	−64.73 (−53.36–−73.33)	Decreasing	−10.97 (−9.19–−12.76)
Death	Stationary	NA	Stationary	NA
**Northeast**				
Notification	Decreasing	−4.92 (−1.23–−6.89)	Stationary	NA
Cure	Decreasing	−19.33 (−16.42–−24.35)	Decreasing	−22.07 (−17.52–−26.36)
Treatment abandonment	Stationary	NA	Decreasing	−3.15 (−0.49–−5.74)
Death	Stationary	NA	Decreasing	−5.62 (−2.89–−8.27)
**South**				
Notification	Decreasing	−5.42 (−1.66–−10.89)	Stationary	NA
Cure	Decreasing	−31.32 (−30.45–−37.56)	Decreasing	−19.52 (−15.15–−23.67)
Treatment abandonment	Stationary	NA	Decreasing	−14.03 (−9.75–−18.09)
Death	Stationary	NA	Stationary	NA
**Southeast**				
Notification	Decreasing	−5.70 (−2.47–−9.39)	Stationary	NA
Cure	Decreasing	−13.12 (−7.52–−18.78)	Decreasing	−21.62 (−16.36–−26.79)
Treatment abandonment	Stationary	NA	Decreasing	−16.11 (−11.04–−20.89)
Death	Stationary	NA	Decreasing	−2.63 (−1.64–−3.60)
**Midwest**				
Notification	Decreasing	−15.35 (−12.53–−18.46)	Decreasing	−2.73 (−1.19–−4.25)
Cure	Decreasing	−60.20 (−30.91–−77.07)	Decreasing	−18.91 (−15.66–−22.03)
Treatment abandonment	Stationary	NA	Stationary	NA
Death	Stationary	NA	Stationary	NA

Legend: 95%CI: 95% confidence interval; MPC: monthly percent change.

## Data Availability

Information regarding TB cases was obtained through the Information Technology Department of the Unified Health System (DATASUS—accessed at: https://datasus.saude.gov.br/, accessed on 26 July 2022). Information on COVID-19 cases was obtained from the Panel Coronavirus (accessed at: https://covid.saude.gov.br/, accessed on 26 July 2022), and population data were extracted from the Brazilian Institute of Geography and Statistics (IBGE—accessed at: https://www.ibge.gov.br/, a accessed on 26 July 2022).
